# Enhancing $H_{2}O_{2}$ Generation Using Activated Carbon Electrocatalyst Cathode: Experimental and Computational Insights on Current, Cathode Design, and Reactor Configuration

**DOI:** 10.3390/catal15020189

**Published:** 2025-02-18

**Authors:** Maria del Mar Cerrillo-Gonzalez, Amir Taqieddin, Stephanie Sarrouf, Nima Sakhaee, Juan Manuel Paz-García, Akram N. Alshawabkeh, Muhammad Fahad Ehsan

**Affiliations:** 1 Department of Chemical Engineering, Faculty of Sciences, University of Malaga, 29010 Malaga, Spain;; 2 Department of Mechanical and Industrial Engineering, Northeastern University, Boston, MA 02115, USA;; 3 Department of Civil and Environmental Engineering, Northeastern University, Boston, MA 02115, USA;

**Keywords:** water treatment, electrocatalysis, GAC, H_2_O_2_ generation, reactor configuration, Bayesian optimization, numerical simulations

## Abstract

Granular activated carbon (GAC) serves as a cost-efficient electrocatalyst cathode in electrochemical water treatment. This study investigates the impact of current intensity and cathode mesh size on the electrocatalytic generation of reactive oxygen species (ROS), i.e., hydrogen peroxide $\left(H_{2}O_{2}\right)$ and hydroxyl radicals (•OH), for removing p-nitrophenol (PNP) as a representative contaminant. The findings suggest that these parameters exert a factorial effect on PNP removal, which is statistically endorsed via the analysis of variance. The −20 + 40 mesh GAC exhibited superior electrocatalytic performance due to its optimal balance of porosity and active surface area. Additionally, the reactor configuration was also studied. Employing two reactors in series configuration resulted in a 23% increase in $H_{2}O_{2}$ generation and a 32% enhancement in overall PNP removal compared with the single reactor configuration. This enhancement is attributed to (i) the enhanced electroactive area, (ii) the greater retention time of PNP over the electrocatalyst surface, and (iii) the increased dissolved oxygen and $H_{2}O_{2}$ content in the second reactor, promoting the overall $H_{2}O_{2}$ generation. Numerical simulations were conducted to compute $H_{2}O_{2}$ concentration profiles, providing a detailed representation of the physical, chemical, and electrochemical processes. The model exhibited a high degree of accuracy compared with the experimental measurements, with $R^{2}$ values ranging from ~0.76 to 0.99 and MAE values between ~0.04 and 0.23 mg/L. The simulation results highlight a strong interplay between $H_{2}O_{2}$ generation, its reaction kinetics during PNP removal, and electrode utilization efficiency. These findings emphasize the importance of optimizing the applied current magnitude and reactor operation duration to maximize electrode efficiency and $H_{2}O_{2}$ generation and utilization, while minimizing electrochemical bubble blockage. Overall, this study provides fundamental insights to optimize the electroactive area for enhanced ROS generation toward efficient contaminant removal, supporting sustainable groundwater remediation technologies in the face of emerging pollutants.

## 1. Introduction

Groundwater contamination is a pressing concern due to the growing prevalence of emerging pollutants originating from industrial, agricultural, and domestic sources [1]. These contaminants include persistent organic pollutants (POPs), pharmaceuticals, per- and polyfluoroalkyl substances (PFAS), and other hazardous chemicals that pose significant risks to human health and aquatic systems [2–5]. Prolonged exposure to these contaminants can result in severe health issues, including respiratory, immunological, and neurological disorders, cancer, and complications during pregnancy [6]. Given the persistence and toxicity of these contaminants, the removal of hazardous substances from wastewater and natural water sources using effective remediation strategies is imperative and urgently needed [7]. Conventional water treatment methods, such as adsorption, chemical oxidation, and biological degradation, often struggle to effectively remove these pollutants, leading to the need for novel, efficient, and sustainable approaches [8].

In contrast, electrochemical advanced oxidation processes (EAOPs) have emerged as promising alternatives due to their versatility and ability to degrade a wide range of contaminants [9,10]. These processes offer several advantages, including the on-site generation of reactive oxygen species (ROS), modular reactor design, effective and optimized operating chemical conditions, and potential energy recuperation, making them highly suitable for decentralized water treatment applications [11]. Specifically, their ability to electrochemically generate and dynamically control various ROS in single system, such as hydrogen peroxide $\left(H_{2}O_{2}\right)$ and hydroxyl radicals (•OH) has emerged as one of the most promising approaches and cost-effective solutions for water treatment [12–14]. Increasing the efficiency of in situ $H_{2}O_{2}$ generation employing such electrochemical reactors can lead to powerful contaminant degradation [2]. One of the most critical factors influencing the performance and efficiency of electrochemical water treatment systems is the reactor design (e.g., batch vs. flow-through regimes and the design of the electrode materials) [15,16]. Several studies have evaluated the effectiveness of various reactor geometries and electrode designs for improving the application of electrochemical water treatment processes [17]. While batch reactors offer controlled conditions for studying electrochemical reactions, these systems are often limited by mass transfer constraint and the gradual depletion of reactants, making them less suitable for long-term applications [17,18].

On the other hand, flow-through electrochemical reactors provide a continuous supply of fresh electrolyte, allowing simultaneous adsorption and contamination degradation, which enhances both reaction efficiency and long-term operational viability [17,18]. In these reactors, multiple physical, chemical, and electrochemical processes are interdependently coupled, including electrochemical reactions, gas evolution, convection transport, adsorption, consumption of reactants, and contaminant degradation [19,20]. A systematic investigation of the coupling between these processes to optimize the degradation efficiency of contaminants is inevitably necessary. Activated carbon (AC)-based electrodes have gained significant attention as a cost-efficient cathode material due to theirhigh surface area, intrinsic adsorption capacity, and improved electrode stability under cathodic conditions. These characteristics facilitate the electrochemical $H_{2}O_{2}$ formation via 2 electron oxygen reduction reaction (2e-ORR), and facilitate the indirect oxidation of persistent contaminants [16,21]. In contrast, when employed as an anode, carbon oxidation might lead to the structural degradation of GAC [22]. Understanding and tuning the coupled processes in flow-through reactors with AC-based electrodes is important to maximize pollutant removal through ensuring synergy between the adsorption and electrochemical transformation of the reactants and contaminants. The efficiency of the electrochemical generation of $H_{2}O_{2}$ depends on multiple factors, including electrode material, AC particle size, and reactor configuration [2]. Previous studies have demonstrated that GAC size affects mass transfer and adsorption capacity [22–25], while reactor configuration, such as employing multi-stage reactor systems (e.g., two in-series reactors), can significantly enhance ROS production, contaminant removal, and pollutant degradation efficiency [26,27]. However, there remains a gap in the understanding of the interplay between these parameters, particularly in flow-through reactors where continuous operation necessitates an optimized balance between electrode utilization, adoption, and electrochemical energy conversion.

This study aims to address this gap by systematically investigating the influence of current density, AC particle size, and reactor configuration on ROS generation and contaminant removal in electrochemical reactors. In particular, we evaluated the performance of single versus two in-series reactors under various conditions to determine the optimal operation conditions for long-term applications. First, experimental measurements were conducted to monitor ROS generation and transport, particularly $H_{2}O_{2}$, under various electrochemical conditions. Next, a computational model was developed to simulate the generation and transport of $H_{2}O_{2}$ concentration profiles, dynamically accounting for the electrode efficiency, active area, reaction kinetics, and mass transport limitations enabling a realistic representation of the reactor. Numerical optimization was performed to determine key parameters, achieving a strong agreement with the experimental data and providing insights into the interplay between $H_{2}O_{2}$ generation, utilization, and electrode performance. By bridging both the experimental and computational findings, this paper provides new insights into the optimization of in situ electrochemical ROS generation and contaminant removal using flow-through reactors. These insights, along with the parametric investigations, will contribute to the development of more efficient and sustainable electrochemical water treatment systems, facilitating practical deployment for long-term groundwater remediation in the face of emerging pollutants and achieving a healthier environment.

## 2. Results and Discussion

### 2.1. Experimental Results

#### 2.1.1. Effect of Current and AC Particle Size

**Figure 1.**
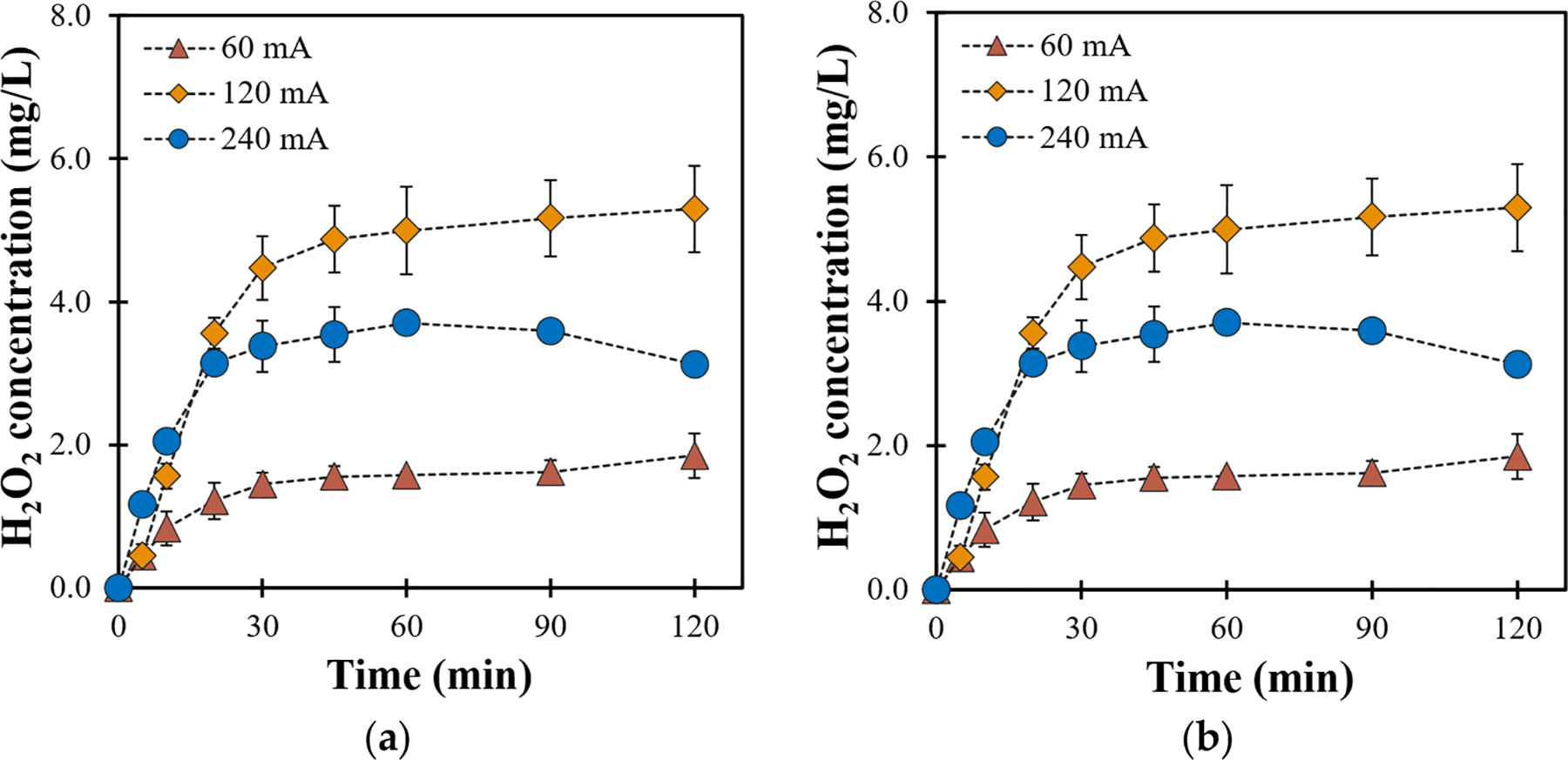
Effect of current on production of (**a**) $H_{2}O_{2}$ and (**b**) •OH (conditions: (−20 + 40) GAC; $3\;\text{mL}{\;\text{min}}^{-1}$).

Figure 1 illustrates the effect of current on $H_{2}O_{2}$ generation and its catalytic decomposition into •OH using a (−20 + 40) mesh GAC electrocatalyst cathode. Increasing the current from 60 to 120 mA results in the $H_{2}O_{2}$ concentration rising from 1.84 to $5.29\;\text{mg}\;L^{-1}$ after 90 min, while at 240 mA it decreased to $3.70\;\text{mg}\;L^{-1}$. This indicates that 2e-ORR is favored at moderate current intensities (e.g., 120 mA) promoting $H_{2}O_{2}$ generation, while higher currents promote 4e-ORR to form $H_{2}O$ instead of $H_{2}O_{2}$, as observed and reported in our previous work [16].

Elevated currents also exacerbate electrowetting effects due to increased gas bubble formation, which obstructs the electrode surface and reduces the effective electroactive area [28]. A higher current (240 mA) results in enhanced oxygen evolution at the anode which oversaturates the GAC surface via the accumulation of oxygen bubbles underneath the cathode causing electrowetting and a reduced electroactive area for $H_{2}O_{2}$ formation, which could also explain the decline in •OH concentration after 45 min (refer to Figure 1b).

**Figure 2.**
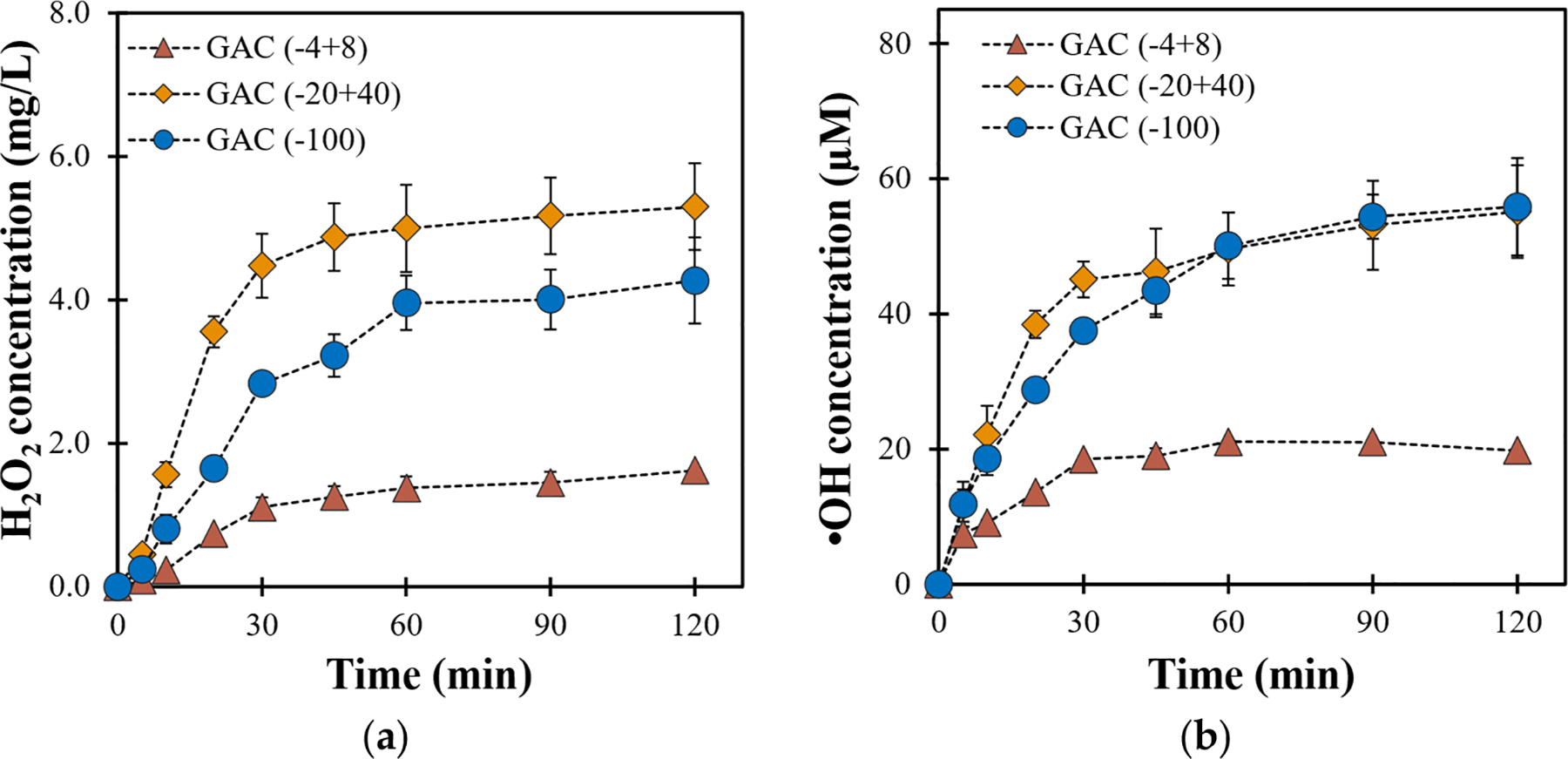
Effect of GAC particle size on production of (**a**) $H_{2}O_{2}$ and (**b**) •OH (conditions: 120 mA; ${3\;\text{mL}\;\text{min}}^{-1}$).

The effect of GAC particle size on electrocatalytic $H_{2}O_{2}$ generation and its catalytic decomposition into •OH was also investigated at 120 mA (Figure 2). The results showed an increase in $H_{2}O_{2}$ concentration from 1.37 to $5.16\;\text{mg}{\;L}^{-1}$ as the GAC mesh size was changed from (−4 + 8) to (−20 + 40), while it decreased to $4\;\text{mg}\;L^{-1}$ with (−100) mesh GAC. These findings can be attributed to GAC porosity and the active surface area associated with a specific mesh size. The porous structure of GAC facilitates the adsorption of dissolved oxygen, which is crucial for 2-eORR. The larger particles in (−4 + 8) mesh GAC favor the permeation of water molecules with dissolved reactants (anodic $O_{2}$ and $H^{+}$) through the electrode. However, the active surface area is lower in (−4 + 8) mesh GAC due to the large particle size. As the GAC particle size decreased, in the case of −20 + 40 mesh GAC, $H_{2}O_{2}$ formation was enhanced due to the increase in the active surface area. Further reduction in GAC particle size using a (−100) mesh sieve decreased the accumulated $H_{2}O_{2}$ concentration, likely due to reduced porosity, which limits the efficient reactant permeation and interaction with the cathode. Additionally, the smaller particle size makes cathode fabrication more challenging, as it is more prone to leaching into the solution thus hampering $H_{2}O_{2}$ generation.

The catalytic decomposition of $H_{2}O_{2}$ to generate •OH (Figure 2b) displayed a similar trend, with the highest •OH concentration (55.82μM) observed for both (−20 + 40) and (−100) mesh AC, indicating optimal porosity and active surface area. Conversely, (−4 + 8) mesh GAC exhibited significantly reduced •OH concentration (20μM). These results highlight the necessity of an optimum GAC size for enhanced in situ ROS generation.

**Figure 3.**
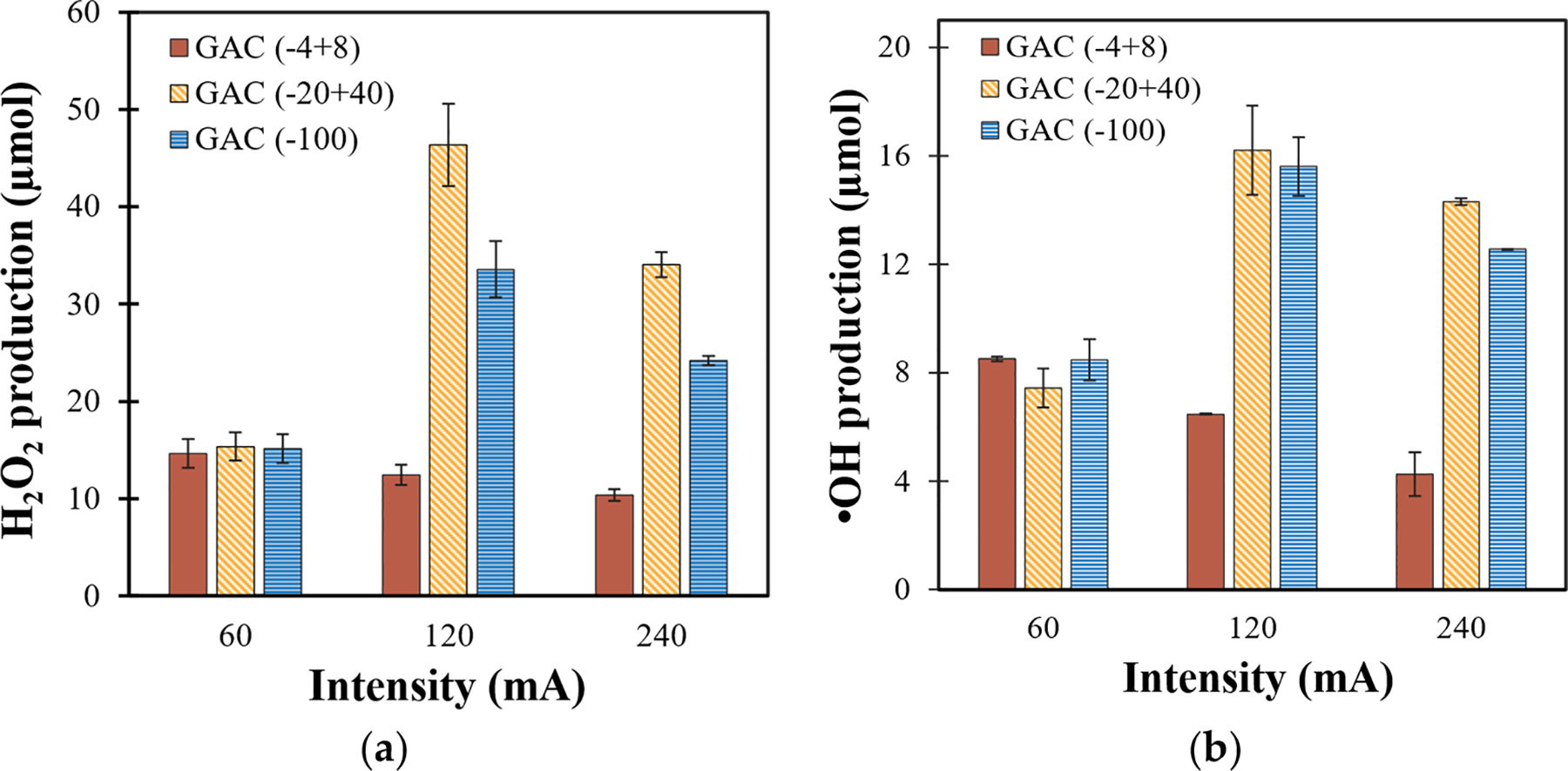
Effect of GAC particle size and current on total production (μmol) of (**a**) $H_{2}O_{2}$ and (**b**) •OH (conditions: $3\;\text{mL}{\;\text{min}}^{-1},$ 2 h).

**Figure 4.**
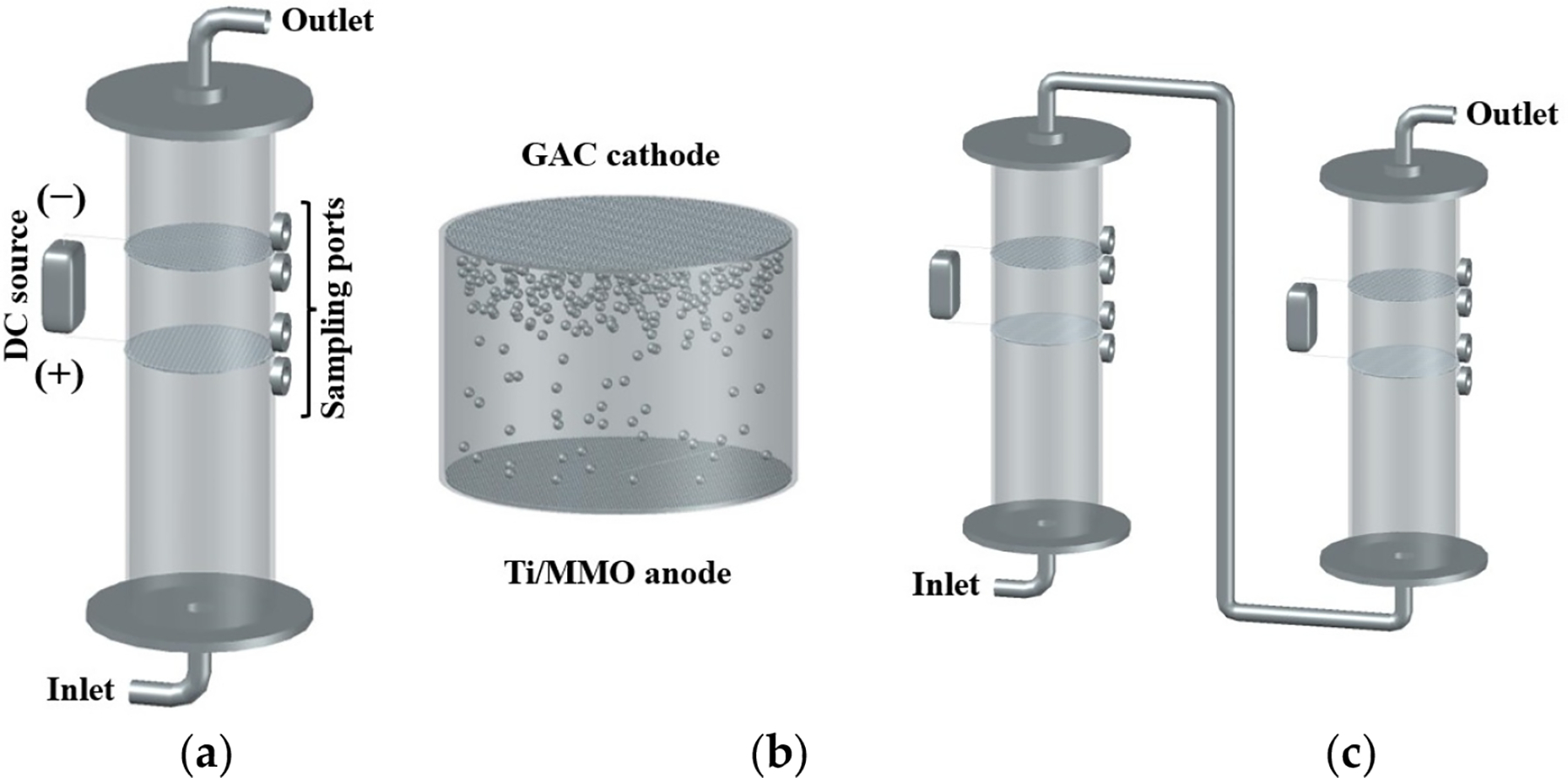
Illustrations for (**a**) single electrochemical flow-through reactor configuration, (**b**) electrowetting phenomenon, and (**c**) in-series reactors configuration.

To better understand the interaction of both these parameters, the co-relation between current and GAC particle size on $H_{2}O_{2}$ generation and its subsequent decomposition into •OH was investigated. The total ROS production was calculated following Equation (1), and the results presented in Figure 3 are summarized in Tables S1 and S3. It can be observed that as the current increases from 60 to 240 mA for (−4 + 8) mesh GAC, $H_{2}O_{2}$ and •OH generation decreases due to $O_{2}$ reduction into water via 4e-ORR and the electrowetting effect. This phenomenon, represented in Figure 4b, involves the accumulation of oxygen bubbles underneath the cathode due to its negligible surface area, hence limiting oxygen adsorption and resulting in decreased ROS formation.

Conversely, the formation of both $H_{2}O_{2}$ and •OH increases as the current increases from 60 to 120 mA for either (−20 + 40) or (−100) mesh GAC. This increase could be attributed to the higher active surface area provided by these smaller particle sizes, which facilitates the adsorption of a higher concentration of dissolved anodic oxygen at moderate current intensities. Dissolved oxygen is essential for 2-eORR to produce $H_{2}O_{2}$. The (−20 + 40) mesh GAC offers an optimal balance of porosity and surface area, allowing effective oxygen diffusion and adsorption. The (−100) mesh GAC, despite having an even better surface area due to its smaller particle size, suffers from reduced porosity and potential GAC leaching, which negatively impacts the ROS generation capacity. However, as the current is further increased to 240 mA, ROS generation decreases due to parasitic reactions and electrowetting at high currents [16,28].

Interestingly, the total •OH production was similar (~16μmol) for both (−20 + 40) and (−100 mesh) mesh GAC, suggesting that the catalytic decomposition of $H_{2}O_{2}$ to •OH may be less influenced by particle size. However, $H_{2}O_{2}$ production was significantly higher for (−20 + 40) mesh GAC (46.36μmol) compared with (−100) mesh GAC (33.57μmol). This highlights the importance of porosity for maintaining efficient oxygen transport and interaction with the cathode surface. Additionally, regardless of GAC size, both $H_{2}O_{2}$ and •OH production decreased at elevated currents (240 mA) due to the dominance of 4e-ORR and the detrimental effects of electrowetting, which further emphasizes the need to optimize current intensity for effective ROS generation.

Analysis of variance (ANOVA) was performed to assess the statistical significance of the influence of the studied variables (current and AC particle size) on ROS generation. The results of these ANOVAs are available in the Supplementary Material (Tables S2 and S4). With a very small statistical probability (p<0.01), ANOVA supports the results from Figure 3, indicating a factorial effect of both current and GAC mesh size on ROS generation. These results suggest that (−20 + 40) GAC and 120 mA current represent the optimum conditions for generating the highest ROS concentrations, which were subsequently employed for the removal of PNP ($5\;\text{mg}\;L^{-1}$) from simulated groundwater.

#### 2.1.2. Reactor Configuration

**Figure 5.**
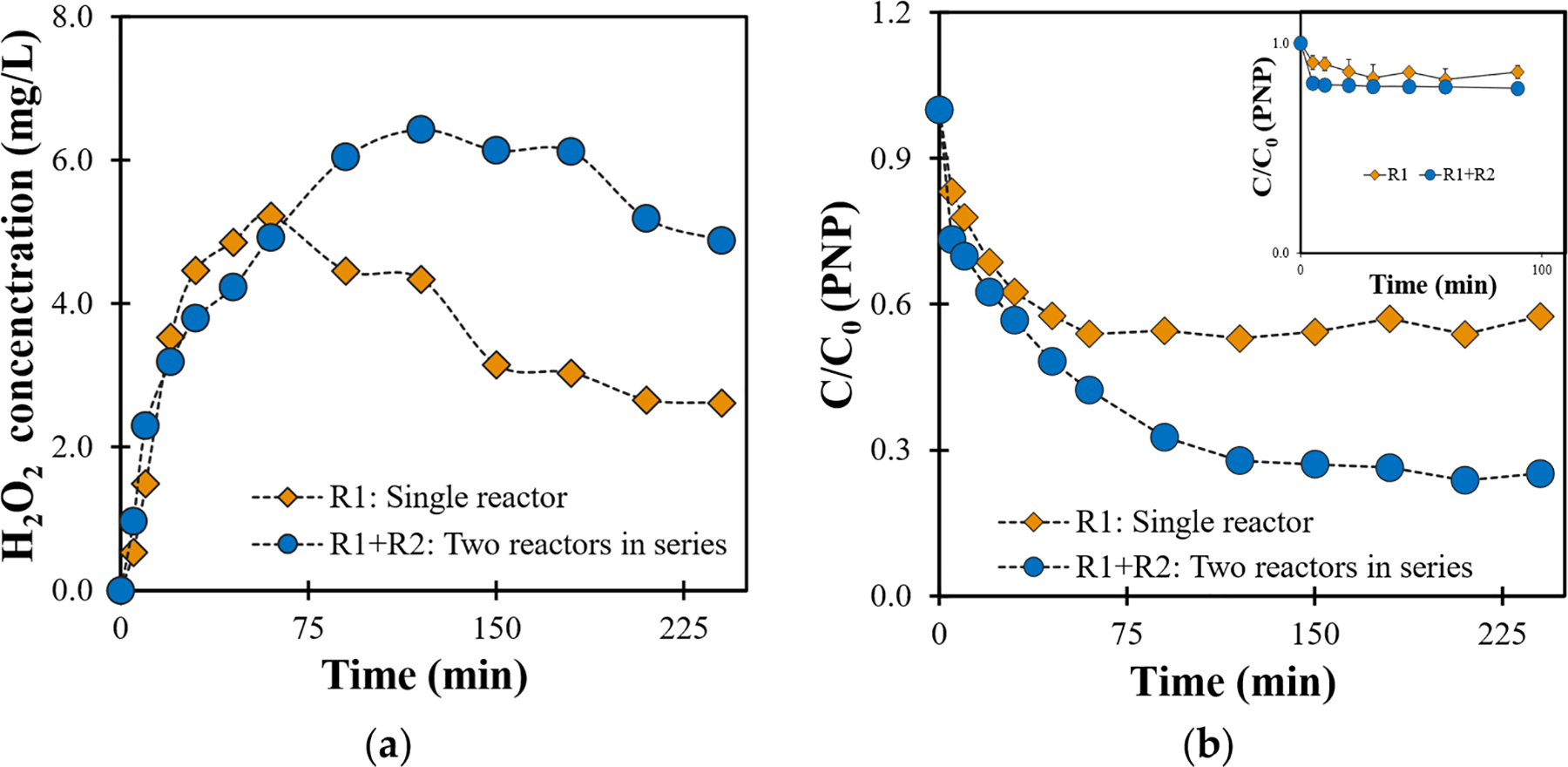
Comparison of single (R1) and two in-series reactors (R1 + R2) on (**a**) $H_{2}O_{2}$ generation and (**b**) PNP removal and adsorption (inset) (conditions: 120 mA; −20 + 40 mesh GAC; $[\text{PNP}{]}_{0}=5\text{mg}\;L^{-1};\;{3\;\text{mL}\;\text{min}}^{-1}$).

The reactor configuration was also evaluated, employing two in-series reactors each using a Ti/MMO anode and a −20 + 40 mesh GAC cathode for the electrochemical removal of PNP. With this reactor configuration, presented in Figure 4c, the effluent from the first reactor served as the influent for the second reactor, allowing for the simultaneous analysis of samples from both reactors at various time intervals. The findings demonstrated that the utilization of this reactor configuration resulted in a 23% enhancement for in situ $H_{2}O_{2}$ generation (Figure 5a) and a 32% improvement in overall PNP removal compared with a single reactor (Figure 5b). This can be attributed to the higher dissolved oxygen content in the additional reactor, leading to increased $H_{2}O_{2}$ generation, as well as the longer contact time between PNP and the electrocatalyst surface and generated ROS in the second reactor. The utilization of such a configuration proved effective in optimizing ROS generation and facilitating the removal of PNP, contributing to the development of efficient electrochemical remediation methods.

Compared to prior reports from the literature (Table 1), the two in-series reactor system demonstrated a competitive performance. While batch reactor and alternative electrode materials can sometimes yield higher ROS accumulation, the in-series flow-through reactor design primarily provides a higher electroactive area that presents advantages in continuous operation with an upscaling potential, making it a promising strategy for electrochemical wastewater treatment applications.

#### 2.1.3. Physiochemical Analysis of −20 + 40 Mesh-Sized GAC

**Figure 6.**
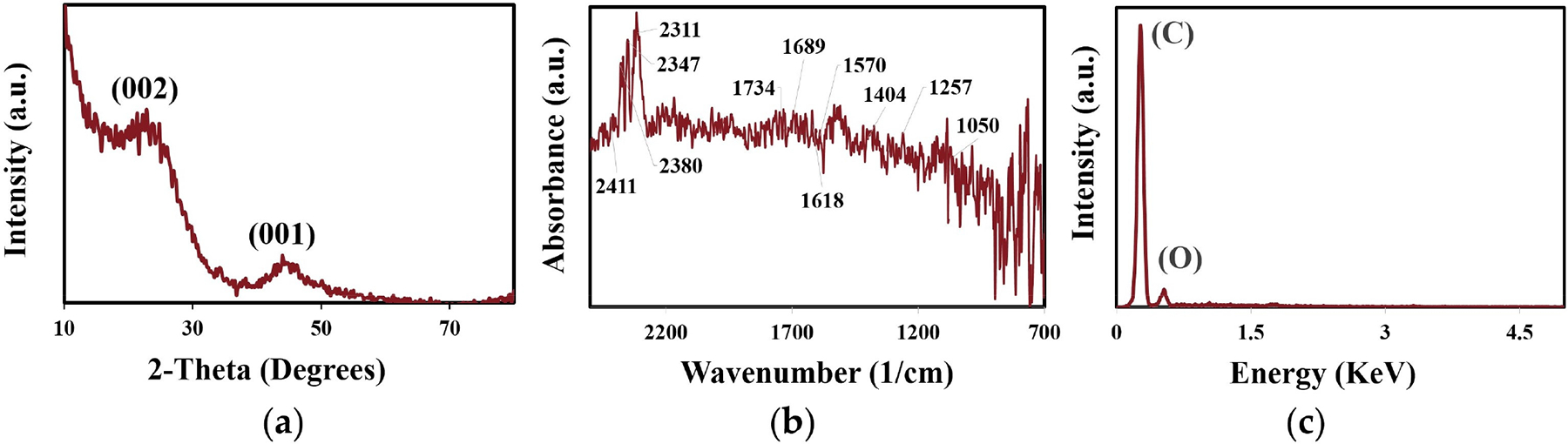
(**a**) XRD, (**b**) FTIR, and (**c**) EDX analysis of the −20 + 40 AC.

**Figure 7.**
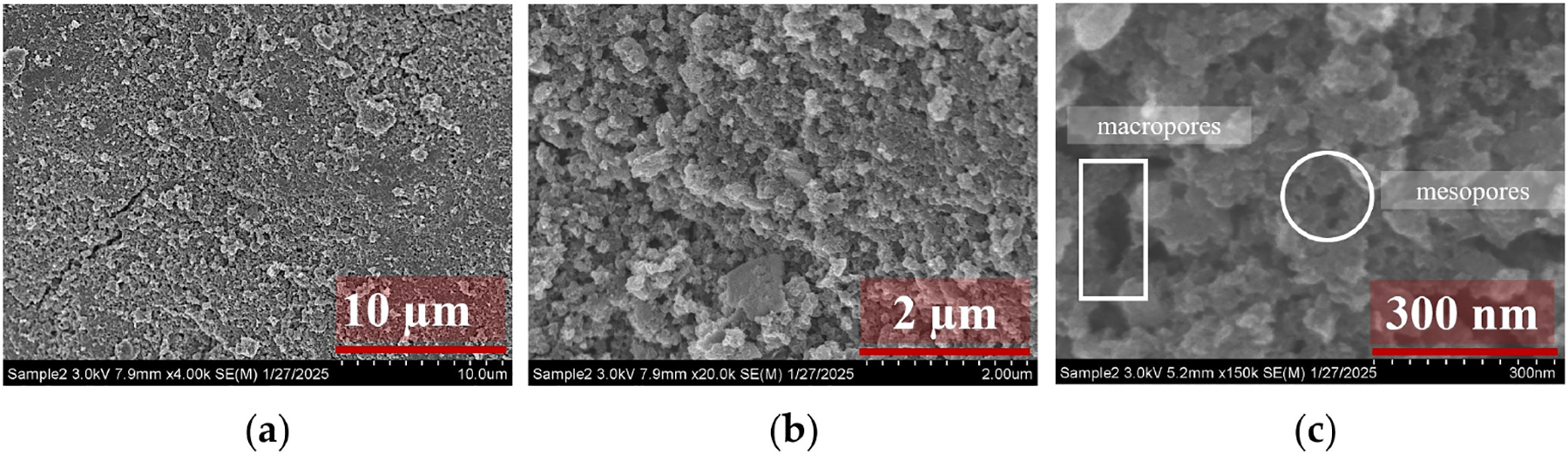
SEM micrographs of −20 + 40 AC at (**a**) 10μm, (**b**) 2μm, and (**c**) 300 nm where the rectangular and circular regions represent the macropores and mesopores areas, respectively.

Based on the electrochemical results using various mesh-sized AC cathodes for ROS generation in a flow-through reactor, −20 + 40 mesh AC exhibited promising performance and was further analyzed for its phase (Figure 6a), surface functionalities (Figure 6b), composition (Figure 6c), and morphology (Figure 7). XRD analysis (Figure 6a) revealed two characteristic peaks at 23° and 45° (2θ), corresponding to the (002) and (101) planes, respectively, of the graphitic hexagonal structure of activated carbon [30]. The broad X-ray diffractogram indicates its amorphous nature, which is a characteristic AC feature. FTIR spectra (Figure 6b) displayed signals corresponding to various surface functionalities influencing $2e^{-}$ ORR for $H_{2}O_{2}$ generation and its subsequent decomposition into •OH. Peaks between 2450 and $2300{\;\text{cm}}^{-1}$ were attributed to alkyne stretching under ambient conditions or adsorbed atmospheric ${\text{CO}}_{2}$ [31]. Absorptions in the $1800-1500{\;\text{cm}}^{-1}$ range were associated with carbonyl functional groups from carboxylic acids, aldehydes, or ketones [32]. A peak at $1404{\;\text{cm}}^{-1}$ indicated C–O–C vibrations of epoxy or alkoxy groups, while peaks in the $1300-1000{\;\text{cm}}^{-1}$ range corresponded to carboxyl groups via carbonyl stretching [33]. Energy-dispersive X-ray spectroscopy (EDX) (Figure 6c) confirmed the purity of the AC, with peaks only attributing to carbon and oxygen. The surface morphology of −20 + 40 mesh AC was also analyzed using a Hitachi 4800 field-emission scanning electron microscope (FESEM), with the results shown in Figure 7. At 10μm magnification (Figure 7a), the AC surface exhibited a uniform rough texture with cracked pores of varying sizes, which was further confirmed at 2μm magnification (Figure 7b). The high-magnification SEM image (300 nm, Figure 7c) clearly revealed the presence of mesopores (circled), while rectangular marks indicated the presence of macropores, providing comprehensive insight into the high-surface-area structure and pore-size distribution that facilitate electrocatalytic reactions at the AC cathode surface.

### 2.2. Simulation Results

**Figure 8.**
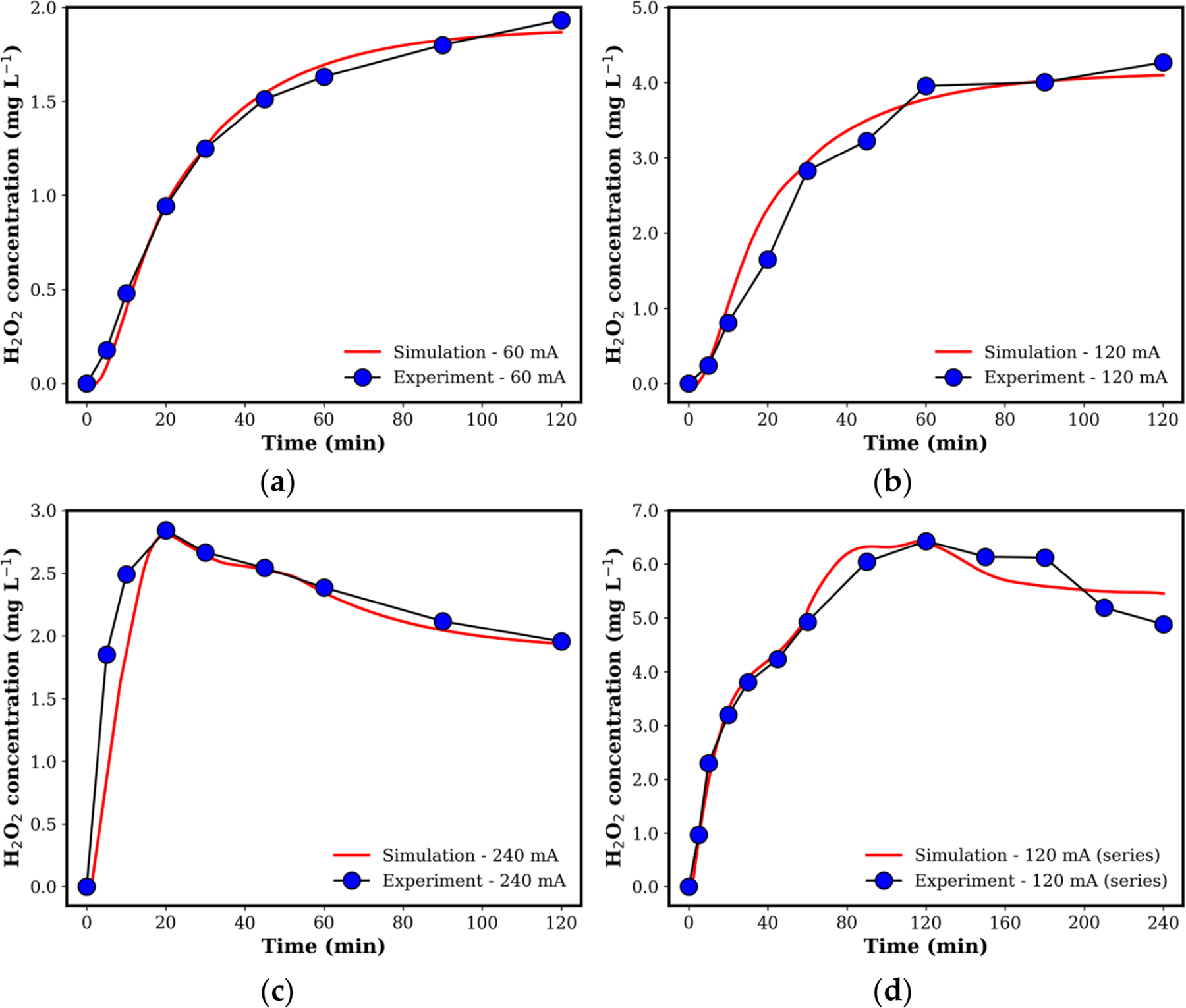
Comparison of experimentally measured and computationally simulated $H_{2}O_{2}$ concentrations for (**a**) 60 mA (single reactor), (**b**) 120 mA (single reactor), (**c**) 240 mA (single reactor), and (**d**) 120 mA (two in-series reactors).

Computational simulations were performed to support and elucidate the experimental results. A numerical framework was developed to capture the generation and transport of $H_{2}O_{2}$ (see Section 3 for more details). The performance and accuracy of the model were evaluated at different current intensities and reactor configurations as summarized in Table 2. Overall, the obtained $H_{2}O_{2}$ concentration profiles as a function of time show excellent agreement with the experimental results for the various studied cases as shown in Figure 8. To better understand the dynamics of $H_{2}O_{2}$, the optimized efficiency factor and reaction rate constant (see Table 2) are used to fundamentally explain the utilization of $H_{2}O_{2}$. At a low current, 60 mA, the computational results show the lowest error (RMSE = 0.052 mg/L and $R^{2}=0.99$), indicating that $H_{2}O_{2}$ generation and transport is well governed by single-phase diffusion and convection processes as modeled. This indicates that the electrode overpotential and side reactions (i.e., oxygen bubble formations) are minimal at this low current. The optimized value of reaction constant k was moderated (i.e., $k=0.229{\;\text{cm}}^{1.584}{\;\text{mol}}^{-0.528}s^{-1}$), indicating limited reactivity between $H_{2}O_{2}$ and/or ROS. Additionally, the optimization of the model shows a low efficiency (i.e., $\theta =0.167{\;\text{cm}}^{-2}$) at this current which suggests that the electrode surface is not fully activated or has not overcome the activation barriers for $H_{2}O_{2}$ generation. Overall, since the reaction kinetics of PNP degradation are slow at low currents, the consumption of $H_{2}O_{2}$ by PNP was minimal, resulting in a stable concentration profile over time (see Figure 8a).

For a medium current intensity, 120 mA, the efficiency improved significantly ($\theta =0.265{\;\text{cm}}^{-2}$), reflecting the enhanced electrode utilization and better reaction kinetics. This indicates that the effective activated surface of the cathode is enhanced at higher current values. At this current, the value of k increased to $0.264{\;\text{cm}}^{1.584}{\;\text{mol}}^{-0.528}{\;s}^{-1}$, which correlates with a faster and more efficient electrochemical generation of $H_{2}O_{2}$. Specifically, the increased rate and concentration of $H_{2}O_{2}$ (Figure 8b) leads to larger consumption of $H_{2}O_{2}$ by PNP. This suggests a more pronounced competition between the reaction kinetics for PNP degradation and $H_{2}O_{2}$ generation at this current intensity compared with a lower current value. This interplay and more complicated physiochemical dynamics lead to an increased error between the simulation and the experimental results (RMSE = 0.27 mg/L and $R^{2}=0.97$) compared with the 60 mA case. However, the model still shows strong accuracy indicating that these processes are still well governed by the diffusion and convection effects.

As the current increases to a higher level, 240 mA, the simulation results show that the reactor initially exhibited high efficiency (i.e., $\theta =0.487{\;\text{cm}}^{-2}$), with an elevated k value during the initial stages $\left(k=0.377{\;\text{cm}}^{1.584}{\;\text{mol}}^{-0.528}{\;s}^{-1}\right)$ for a time less than 9.5 min. This indicates a very rapid generation of $H_{2}O_{2}$ and high electrode utilization. However, as the operation progressed, a decline in efficiency was observed over the subsequent periods where θ=0.0925 and $0.061{\;\text{cm}}^{-2}$ between 9.5–41 min and beyond 41 min, respectively. These periods were identified by Bayesian optimization. This drop in efficiency as a function is due to reduced electrode surface availability and increased overpotential caused by oxygen bubble-induced blockage, limiting the system’s ability to sustain high reaction rates [34,35]. This is confirmed by the lower match between the simulation and experiments compared with the previous cases (i.e., the RMSE = 0.39 mg/L and $R^{2}=0.76$ for the 240 mA case), which indicates that high-order interactions are taking place at these elevated currents. Specifically, the single-phase diffusion and convection mechanisms can be limited to fully describing the multi-phase interactions at high current levels. The bubble blockage leads to a lower production of $H_{2}O_{2}$ compared with the previous medium current case (Figure 8b,c). Further, the reaction coefficient k shows higher values compared with the previous case where the k value kept increasing before it saturated at this large current (Table 2). Although $H_{2}O_{2}$ generation is limited at this stage, as indicated by the reduced value of θ, the increase in k over time can be attributed to complex multi-phase chemical interactions between ROS, PNP, and $H_{2}O_{2}$ which require more detailed modeling to reveal.

Finally, the two-electrode series reactor simulation with a current value of 120 mA shows a higher efficiency $\left(\theta =0.463{\;\text{cm}}^{-2}\right)$ at the initial stage (i.e., a period less than 10.4 min) compared with the single reactor with the 120 mA case. Then, θ decreases to $0.271{\;\text{cm}}^{-2}$ before converging to $0.21{\;\text{cm}}^{-2}$ as the time increases. These values over a longer time are within the range of the obtained efficiency using a single reactor. The decrease in the efficiency over time is expected as more bubbles accumulate at the electrode surface causing larger overpotential and lower utilization of the electrode [34,35]. Despite this, the model shows that the processes are still well governed by single-phase diffusion and convection given that the simulation results are very close to the experimental results (i.e., RMSE = 0.29 mg/L and $R^{2}=0.98$). These results, along with the increased concentration of $H_{2}O_{2}$ (Figure 8d) compared with the single-reactor configuration, show that using two reactors allowed for an improved distribution of current density, reducing localized bubble formation and sustaining $H_{2}O_{2}$ generation. Specifically, the two in-series reactors configuration demonstrates the advantage of spreading the electrochemical load across multiple electrodes compared with the single 240 mA case. Overall, the experimental and computational results suggest that the system’s performance could be optimized by dynamically adjusting operating conditions. One strategy is to initiate operation at 240 mA to rapidly generate $H_{2}O_{2}$, followed by switching to lower currents or increasing the flow rate to mitigate bubble accumulation. After sufficient bubble removal, the current could be reverted to 120 mA for sustained and efficient $H_{2}O_{2}$ generation and utilization. Additionally, incorporating a two-electrode system with intermediate currents can further enhance efficiency by balancing $H_{2}O_{2}$ generation and its reaction kinetics with PNP removal.

## 3. Methodology

### 3.1. Materials and Chemicals

The chemicals used in this work were of analytical grade. The granular activated carbon (GAC) of −4 + 8 mesh was purchased from Fisher Scientific, Hampton, NH, USA. The sodium sulfate (anhydrous, ≥99%), calcium sulfate (99.9%), and titanium sulfate (99.9%) were obtained from Sigma-Aldrich, St. Louis, MO, USA. The benzoic acid was acquired from Fisher Scientific and was used for •OH radicals quantification. PTFE (60% Sigma-Aldrich) and ethanol were used to fabricate the cathode. Deionized water was used in all the experiments, and it was obtained from a Millipore Milli-Q system, St. Louis, MO, USA. Stainless steel mesh was used as cathode current collector, while titanium mixed metal oxide (Ti/MMO) mesh electrode was used as the anode for oxygen evolution. The Ti/MMO anode, composed of ${\text{IrO}}_{2}$ and ${\text{Ta}}_{2}O_{5}$ coating on titanium, was cut into circular pieces with a diameter of 4.2 and thickness of 1.8 mm.

### 3.2. Fabrication of Cathodes

The −4 + 8 mesh activated carbon (AC) was washed with DI water and dried at 70 °C for 12 h. The pretreated −4 + 8 GAC with documented particle size of >2380μm was further ground and sieved using −20 + 40 and −100 mesh sieves, resulting in two additional granulometry classes with a particle size ranging from 841 to 400μm and <150μm, respectively. The fabrication of each cathode was carried out by mixing 1.5 g of GAC with a PTFE/ethanol emulsion (1:3v/v). The suspension was vortexed for 5 min. Once the mixture was well dispersed, it was applied to a stainless steel mesh current collector (∅4.3cm) and annealed at 350 °C for 1 h in a muffle furnace. Based on the GAC mesh size, the electrodes were labeled as −4 + 8 GAC, −20 + 40 GAC, and −100 GAC, respectively.

### 3.3. Electrochemical Test

All tests were performed in a flow-through electrochemical reactor as represented in Figure 4a. The reactor is a vertical acrylic cylinder with an inner diameter of 4.3 cm and length of 15 cm. There are four sampling ports available, and we used two of them: one at 3 cm and the other at 6 cm from the top. The electrode configuration used was the anode at the upstream and the cathode at the downstream, with the two components 3 cm apart from each other. The influent was injected at the bottom of the reactor, going firstly through the anode, oxidizing water to generate oxygen, and secondly through the cathode, reducing the anodic oxygen to $H_{2}O_{2}$ and its simultaneous decomposition to generate •OH. The relevant chemical reactions have previously been reported [2,16,22,28]. Finally, the solution exited the reactor at the top. In the case of the two reactors set up in series, effluent from the first reactor was the influent in the second reactor, as represented in Figure 4c. The flow rate was fixed for all the tests at 3 mL/min. A constant current (60, 120, and 240 mA) was supplied by an Agilent E3612A power supply. The applied potential was not controlled directly but it varied to maintain the fixed current.

The experiments were carried out using simulated groundwater as the electrolyte with a concentration of 3 mM of ${\text{Na}}_{2}{\text{SO}}_{4}$ and 0.5 M of ${\text{CaSO}}_{4}$. Benzoic acid (10 mM) was added to the electrolyte to quantify the production of hydroxyl radicals. Moreover, contaminant removal experiments were conducted using PNP (5 ppm) as a model pollutant using different reactor configurations. The conditions of the different tests are detailed in Table 3.

Samples were collected at different times during the experiments and chemically analyzed. The concentration of $H_{2}O_{2}$ was determined using the titanium sulfate method at 405 nm on a Shimadzu UV–Vis spectrometer [8]. The quantification of hydroxyl radicals was carried out following the formation of 4-hydroxybenzoic acid which was analyzed by high-performance liquid chromatography (HPLC) with a UV detector at 254 nm using an Agilent 1260 Infinity Quaternary LC, with a mobile phase of 20% methanol and 80% HPLC water with pH 2–3 adjusted with phosphoric acid. The para-nitrophenol (PNP) concentration was quantified calorimetrically on a Shimadzu UV–Vis spectrophotometer at 400 nm, adding 0.5 mL of 0.1 M NaOH to 3 mL of sample to turn the solution yellow. The total production (μM) of $H_{2}O_{2}$ and OH radicals was calculated according to Equation (1):

(1) $$\text{Total}\;\text{ROS}\;\text{production}=v\int_{t_{o}}^{t}C_{i}\;\text{dt}$$

where t is the reaction time (min), $C_{i}$ is the concentration of $H_{2}O_{2}$ or •OH radicals (μM), and v is the flow rate (mL/min).

### 3.4. Computational Modeling

To support and explain the experimental findings, the generation and transport of $H_{2}O_{2}$ were computationally simulated by solving the Nernst–Planck equation for dilute electrolytes. The flux of $H_{2}O_{2},J_{H_{2}O_{2}}$ via convection and diffusion processes is expressed as

(2) $$J_{H_{2}O_{2}}={\text{uc}}_{H_{2}O_{2}}-D_{H_{2}O_{2}}\nabla c_{H_{2}O_{2}}$$

where $u\left(\text{cm}/s\right)$ is the flow velocity of water, $c_{H_{2}O_{2}}\;\left(\text{mol}{\;\text{cm}}^{-3}\right)$ is the concentration, and $D_{H_{2}O_{2}}$ (${1.42\;\text{cm}}^{2}/s$ [36]) is the diffusion coefficient of $H_{2}O_{2}$ in water. The migration term is excluded (set to zero) as $H_{2}O_{2}$ an uncharged species. The time-dependent conservation $H_{2}O_{2}$ mass is governed by

(3) $$\frac{\partial c_{H_{2}O_{2}}}{\partial t}=-\nabla J_{H_{2}O_{2}}+R_{H_{2}O_{2}}$$

where t is the time, and $R_{H_{2}O_{2}}$ is the bulk reaction term of $H_{2}O_{2}$ representing the consumption and formation of $H_{2}O_{2}$ due to reactions with other species such as PNP or ROS. The reaction term is modeled as

(4) $$R_{H_{2}O_{2}}=-k(t)c^{m}H_{2}O_{2}$$

where $k(t)\;\left({\text{mol}}^{1-m}\;{\text{cm}}^{-(1-m)}\;s^{-1}\right)$ is the reaction constant and m is the reaction order. The reaction constant, k(t), is time-dependent to account for the dynamic changes in the reactant concentrations (e.g., PNP). Both k(t) and m are determined via numerical optimization as discussed later in this section.

Equations (3) and (4) were solved using a one-dimensional finite-difference model representing the electrochemical reactor. Only the cathode was included in the reactor to account for the generation of $H_{2}O_{2}$ via an electrochemical Faradaic reaction. The reactor length and diameter were set to L = 15 and d = 4.3 cm, respectively, consistent with the experimental conditions. The experimental flow rate of water, Q=3mL/min, was used to calculate the flow velocity for the simulation such that

(5) $$u=4Q/\left(\pi d^{2}\right)=3.44\times 10^{-3}\;\text{cm}{\;s}^{-1}$$

The equations were discretized as follows: the time-dependent term was solved using the Runge–Kutta method with fifth-order accuracy with adaptive time control; the advection term employed a first-order upwind scheme; and the diffusion term used a second-order central difference scheme. The spatial domain was discretized into 800 grid points, achieving a numerical error in the order of $10^{-4}$, verified by a numerical convergence analysis (see Figure S1 in the Supplementary Information). An adaptive timestep was used with a maximum of 0.1 s to ensure numerical stability. The equations were iteratively solved at each timestep with an error tolerance of $10^{-6}$. The initial condition of $c_{H_{2}O_{2}}$ was set as

(6) $$c_{H_{2}O_{2}}(x,t=0)=0$$

where x is the spatial dimension such that xϵ[0,L]. Two boundary conditions were used at the inlet and at the electrode surface boundaries as follows, respectively:

(7) $${\left.C_{H_{2}O_{2}}(x=0,t)\right|}_{\text{inlet}}=0$$

(8) $${\left.J_{H_{2}O_{2}}\left(x=x_{\text{electrode}},t\right)\right|}_{\text{electrode}}=\theta I/nF$$

where $x_{\text{electrode}}(=11\;\text{cm})$ is the cathode location, $\theta \left({\text{cm}}^{-2}\right)$ is an efficiency factor that represents the efficiency of the electrochemical reactions per effective area which accounts for the electrode kinetics and wetting [2,34,35], I (A) is applied current, n(=2) is the number of transferred electrons, and $F\left(=96,485\;A\;s{\;\text{mol}}^{-1}\right)$ is Faraday’s constant. The efficiency factor, $\theta \left({\text{cm}}^{-2}\right)$, is presented as normalized by the effective area to address the dynamic changes in the electrode active area over time and with varying applied currents, avoiding oversimplification by assuming a constant effective area across all simulations and cases. The value of θ(t) was determined using numerical optimization as discussed later. Equation (6) represents a flux boundary condition for the generation of $H_{2}O_{2}$ at the electrode where the left-hand side is presented in Equation (2) [34]. The flow velocity was set to zero at the electrode surface for Equation (8) to satisfy the no slip condition (i.e., ${\left.u\right|}_{\text{electrode}}=0$) [37].

For the two in-series reactors system, the total length of the simulation system was doubled such as $L_{\text{total}}=2\times L$ and $x\;\epsilon \;\left[0,L_{\text{total}}\right]$. The spatial domain was discretized using 1600 points to maintain the same numerical accuracy. The second electrode was placed at $x_{\text{electrode}}=26\;\text{cm}$. The boundary condition of Equation (8) was applied at both electrodes (11 and 26 cm) along with the initial in (Equation (6)) and inlet (Equation (7)) conditions for the full system.

#### 3.4.1. Parameter Optimization

Bayesian optimization, implemented using the Optuna package in Python 3.13.2, was employed to determine the values of k(t),m, and θ. A non-grid search of 1000 iterations was used to minimize the root mean square error (RMSE) between the experimental data and the simulation results of $c_{H_{2}O_{2}}$ at a distance of 1 cm from the electrode surface. The RMSE is given as

(9) $$\text{RMSE}=\sqrt{\frac{1}{n}\sum_{i=1}^{n}{\left(c_{H_{2}O_{2},\text{simulated}}-c_{H_{2}O_{2},\text{measured}}\right)}^{2}}$$

where n is the number of data points, $c_{H_{2}O_{2},\text{simulated}}$ is the computationally simulated concentration, and $c_{H_{2}O_{2},\text{measured}}$ is the experimentally measured concentration. The difference between the simulated and measured concentrations is computed for each i data point at a given time. The optimized parameters are reported in Table 2. Initially, the optimization was performed using the measured data for I=60mA to determine k(t),m, and θ(t). The value of m was found to be approximately 1.528 which was then fixed for subsequent optimizations. For other cases, only k(t) and θ(t) were optimized.

#### 3.4.2. Performance Evaluation of the Model

After determining the optimized parameters, the simulations were performed to compute the concentration of $c_{H_{2}O_{2}}$ as a function of time. The RMSE, mean absolute error (MAE), and coefficient of determination $\left(R^{2}\right)$ were calculated to compare the experimental and simulation data. The MAE and $R^{2}$ are defined as

(10) $$\text{MAE}=\frac{1}{n}\sum_{i=1}^{n}\left|c_{H_{2}O_{2},\text{simulated}}-c_{H_{2}O_{2},\text{measured}}\right|$$

(11) $$R^{2}=1-\frac{\sum_{i=1}^{n}{\left(c_{H_{2}O_{2},\text{simulated},i}-c_{H_{2}O_{2},\text{measured},i}\right)}^{2}}{\sum_{i=1}^{n}{\left({\overset{‾}{c}}_{H_{2}O_{2},\text{measured}}-c_{H_{2}O_{2}\text{,measured},i}\right)}^{2}}$$

where ${\overset{‾}{c}}_{H_{2}O_{2},\text{measured}}$ is the mean value of the measured concentration.

## 4. Conclusions

In this study, we investigated the impact of current intensity and granular activated carbon (GAC) particle size on in situ $H_{2}O_{2}$ and •OH generation for PNP removal, followed by an evaluation of reactor configurations using the optimized parameters (120 mA current and −20 + 40 mesh GAC). The experimental results showed that the −20 + 40 mesh GAC electrocatalyst achieved optimal ROS concentrations and enhanced PNP removal at 120 mA. In contrast, elevated currents (240 mA) reduced $H_{2}O_{2}$ and •OH production due to dominated 4e-ORR, which reduces the anodic oxygen directly into $H_{2}O$ rather than supporting 2e-ORR for the electrocatalytic synthesis of $H_{2}O_{2}$. Additionally, the electrowetting effects at higher currents hindered solution–cathode contact, leading to decreased electrode efficiency over time. Using two reactors in series improved $H_{2}O_{2}$ generation by 23% and PNP removal by 32% compared with a single reactor, benefiting from increased dissolved oxygen content, prolonged contact time, and cumulative ROS contributions. Computational simulations further supported these findings, validating 120 mA as the most efficient current for $H_{2}O_{2}$ generation and pollutant removal. Although certain simplifying assumptions, such as constant electrode efficiency, introduced minor deviations at higher currents, the model effectively captured key trends and incorporated a modifying coefficient to account for electrowetting. The computational results show that $H_{2}O_{2}$ generation and transport can be governed by single diffusion and convection processes at current values less than 120 mA for single or multiple reactors. However, applying larger currents leads to more complex dynamics which include multi-phase interactions between gas bubbles, solid electrode, and liquid electrolyte. Further, the computational results along with the experimental observation indicate that the electrode efficiency and utilization can be maximized by applying dynamic operational conditions such as employing variable current. Using the two reactors in series configuration can also enhance the system performance and help to mitigate the bubble-induced overpotentials. Overall, the experimental and computational results emphasize the importance of optimizing current intensity, GAC particle size, and reactor configurations for efficient ROS generation and pollutant removal, contributing to the development of sustainable electrochemical water treatment technologies.

## Supplementary Material

SI

## Figures and Tables

**Table 1.** Comparison of $H_{2}O_{2}$ production and contaminants degradation with the literature.

Electrocatalyst	Cathode Mass (g)	Electrolyte	Current Intensity (mA)	Reactor Configuration	Flow Rate (mL/min)	$H_{2}O_{2}$ (mg/L)	•OH (μM)	Contaminant Degradation (%)	Ref.
GAC (−20 + 40)	1.5	3 mM ${\text{Na}}_{2}{\text{SO}}_{4}$ 0.5 M ${\text{CaSO}}_{4}$	120	Single flow-through reactor	3	5.29	55.82	43% PNP	This study
GAC (−20 + 40)	1.5	3 mM ${\text{Na}}_{2}{\text{SO}}_{4}$ 0.5 M ${\text{CaSO}}_{4}$	120	Two flow-through reactors in series	3	6.43	-	75% PNP	This study
Banana peel biochar	1.5	3 mM ${\text{Na}}_{2}{\text{SO}}_{4}$ 0.5 M ${\text{CaSO}}_{4}$	250	Single flow reactor		3.3	-	41% Ibuprofen	[28]
GAC (−4 + 8) and Fe^2+^ iron	3	10 mM ${\text{Na}}_{2}{\text{SO}}_{4}$ 0.2 mM ${\text{FeSO}}_{4}$	100	Batch reactor	-	20.36	224.53	Inactivate 10^8^ CFU/mL *E.coli*	[21]
AC	10	5 mM ${\text{Na}}_{2}{\text{SO}}_{4}$	100	Single flow reactor	2	-	-	79% PNP	[16]
GAC (−4 + 8)	1.5	50 mM ${\text{Na}}_{2}{\text{SO}}_{4}$	100	Batch reactor	-	8.9	-	61.9% RB19	[14]
Granular bamboo-based biochar	2	50 mM ${\text{Na}}_{2}{\text{SO}}_{4}$	50	Batch reactor	-	11.3	-	72.6% RB19 90.4% Orange II	[29]

**Table 2.** Optimized simulation parameters and evaluation metrics comparing experimental and simulation data across different experimental cases. A reaction order of m=1.528 was used for all cases.

Experiment Case	$\theta \left({\text{cm}}^{-2}\right)$	$k\left({\text{cm}}^{1.584}\;{\text{mol}}^{-0.528}\;{\text{s}}^{-1}\right)$	RMSE (mg/L)	MAE (mg/L)	$R^{2}$
*I* = 60 mA—single	0.167	0.289	0.052	0.041	0.99
*I* = 120 mA—single	0.265	0.264	0.27	0.19	0.97
*I* = 240 mA—single	0.487 for 0 < *t* ≤ 9.5 min, 0.0525 for 9.5 < *t* ≤ 41 min, 0.061 for 41 < *t* ≤ 120 min	0.377 for 0 < *t* ≤ 9.5 min, 0.441 for 9.5 < *t* < 41 min, 0.402 for 41 < *t* ≤ 120 min	0.159	0.25	0.76
*I* = 120 mA—two in-series	0.463 for 0 < *t* ≤ 10.4 min, 0.271 for 10.41 < *t* ≤ 120 min, 0.21 for 120 < *t* ≤ 240 min	0.206 for 0 < *t* ≤ 120 min, 0.114 for 120 < *t* ≤ 240 min,	0.29	0.23	0.98

**Table 3.** Experimental conditions.

Test	GAC Size (Mesh)	Current (mA)	Contaminant	N° of Reactors
A1	−20 + 40	60	10 mM benzoic acid	1
A2	−20 + 40	120	10 mM benzoic acid	1
A3	−20 + 40	240	10 mM benzoic acid	1
A4	−4 + 8	60	10 mM benzoic acid	1
A5	−4 + 8	120	10 mM benzoic acid	1
A6	−4 + 8	240	10 mM benzoic acid	1
A7	−100	60	10 mM benzoic acid	1
A8	−100	120	10 mM benzoic acid	1
A9	−100	240	10 mM benzoic acid	1
A10	−20 + 40	120	5 mg/L 4-nitrophenol	1
A11	−20 + 40	120	5 mg/L 4-nitrophenol	2 (in-series)

## Data Availability

The generated data are available from the corresponding author upon request.
