# Supplementary material for: Enhancing H2O2 Generation Using Activated Carbon Electrocatalyst Cathode: Experimental and Computational Insights on Current, Cathode Design, and Reactor Configuration
Source: Catalysts. Author manuscript; Available in PMC 2025 Sep 24. (PMC12456440; doi:10.3390/catal15020189)
Supplement: SI [file NIHMS2067082-supplement-SI.pdf]

Enhancing H_2O_2 generation using activated carbon electrocatalyst cathode: Experimental and computational insights on current, cathode design, and reactor configuration

Maria del Mar Cerrillo-Gonzalez[1], Amir Taqieddin[2], Stephanie Sarrouf[3], Nima Sakhaee[3], Juan Manuel Paz-García[1], Akram N. Alshawabkeh[3], Muhammad Fahad Ehsan[3,*]

Supplementary data

The following pages include the experimental data, along with its corresponding ANOVA (Tables S1 to S4). The Analysis of variance (ANOVA) comprises of figuring out which factors altogether influence the reaction, utilizing the understudy's t-test.

Table S1. Total production (µmol) of H_2O_2

		Current intensity (mA)		
		60	120	240
GAC size Mesh	-100	16.634	30.673	23.732
	-100	13.69	36.48	24.67
	-20+40	23.25	50.04	32.75
	-20+40	15.74	47.33	35.36
	-4+8	16.09	11.42	10.95
	-4+8	13.15	13.47	9.78

Table S2. ANOVA for H_2O_2: Two-factor with replication, α=0.01

	I (mA)					
SUMMARY	60	120	240	Total		
GAC = -100						
Count	2	2	2	6		
Sum	30.33	67.15	48.40	145.88		
Average	15.16	33.58	24.20	24.31		
Variance	4.32	16.86	0.44	72.14		
GAC = -20+40						
Count	2	2	2	6		
Sum	38.99	97.37	68.11	204.47		
Average	19.50	48.68	34.05	34.08		
Variance	28.15	3.67	3.42	177.44		
GAC = -4+8						
Count	2	2	2	6		
Sum	29.24	24.89	20.73	74.86		
Average	14.62	12.44	10.36	12.48		
Variance	4.33	2.11	0.69	5.05		
Total						

Count	6	6	6			
Sum	98.56	189.41	137.23			
Average	16.43	31.57	22.87			
Variance	13.07	269.61	114.21			
ANOVA						
Source of Variation	*SS*	*df*	*MS*	*F*	*P-value*	*F crit*
Size	1404.3	2	702.13	98.77	7.527E-07	8.02
Current	692.9	2	346.47	48.74	1.4842E-05	8.02
Size-Current	516.2	4	129.06	18.15	2.46E-04	6.42
Within	64.0	9	7.11			
Total	2677.4	17				

Table S3. Total production (μmol) of •OH radicals

		Current intensity (mA)		
		60	120	240
GAC size Mesh	-100	7.705	14.513	12.558
	-100	9.23	16.68	12.53
	-20+40	6.18	16.03	14.19
	-20+40	7.83	14.64	14.43
	-4+8	8.42	6.44	3.45
	-4+8	8.60	6.50	5.06

Table S4. ANOVA for OH radicals: Two-factor with replication, α=0.01

	I (mA)					
SUMMARY	60	120	240	Total		
GAC = -100						
Count	2	2	2	6		
Sum	16.94	31.19	25.09	73.22		
Average	8.47	15.60	12.55	12.20		
Variance	1.16	2.35	0.00	10.94		
GAC = -20+40						
Count	2	2	2	6		
Sum	14.01	30.67	28.61	73.29		
Average	7.00	15.34	14.31	12.22		
Variance	1.36	0.98	0.03	16.98		
GAC = -4+8						
Count	2	2	2	6		
Sum	17.02	12.94	8.51	38.48		
Average	8.51	6.47	4.26	6.41		

Variance	0.02	0.00	1.28	3.89		
Total						
Count	6	6	6			
Sum	47.97	74.80	62.21			
Average	7.99	12.47	10.37			
Variance	1.10	22.26	23.31			
**ANOVA**						
Source of Variation	*SS*	*df*	*MS*	*F*	*P-value*	*F crit*
Size	134.4	2	67.20	84.26	1.4879E-06	8.02
Current	60.1	2	30.04	37.66	4.2389E-05	8.02
Size-Current	91.7	4	22.93	28.75	3.8654E-05	6.42
Within	7.2	9	0.80			
Total	293.4	17				

Table S5. CE (%) of total production of ROS species

	**Intensity**		
**GAC mesh**	**60 mA**	**120 mA**	**240 mA**
(-4+8)	1.03	0.42	0.16
(-20+40)	1.01	1.39	0.54
(-100)	1.05	1.09	0.27

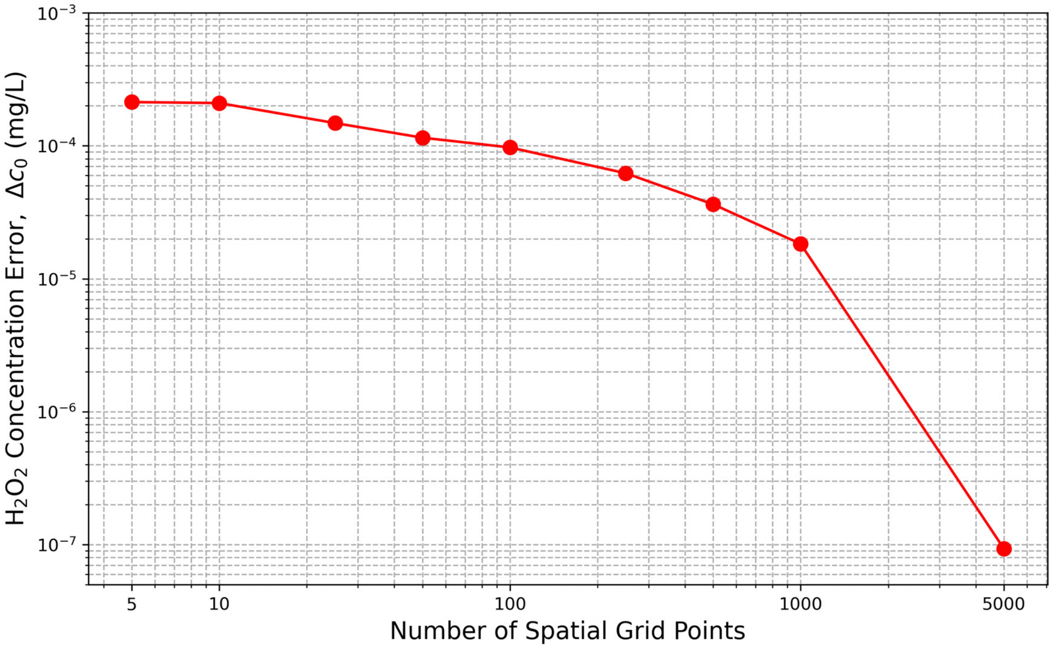

Figure S1. Numerical grid convergence analysis for the simulation. The y-axis represents the algebraic difference between the simulated concentration at the reactor outlet using a given number of spatial grid points and the simulated concentration using 10,000 spatial grid points.
